# Adaptation of Trajectory of Illness Framework to Assess the Experiences of Youths Living with Type 1 Diabetes Mellitus in the Rural Areas of Limpopo Province, South Africa

**DOI:** 10.3390/ijerph23050684

**Published:** 2026-05-21

**Authors:** Thembi Julia Motsepe, Gsakani Olivia Sumbane, Takalani Edith Mutshatshi, Leshata Winter Mokhwelepa

**Affiliations:** 1 Department of Nursing Science, School of Health Care Science, Faculty of Health Science, University of Limpopo, Private Bag X 1106, Sovenga, Polokwane 0727, South Africa; motsepethembi@gmail.com (T.J.M.); takalani.mutshatshi@ul.ac.za (T.E.M.); 2 School of Medicine, Faculty of Health Science, University of Limpopo, Private Bag X 1106, Sovenga, Polokwane 0727, South Africa

**Keywords:** diabetic mellitus, Type 1, youth, experiences, trajectory, illness

## Abstract

**Highlights:**

**Public health relevance—How does this work relate to a public health issue?**

- Explores the lived experiences of youths managing Type 1 Diabetes Mellitus in rural Limpopo province, highlighting challenges in long-term illness management.
- Addresses disparities in access to diabetes care and support services in underserved rural communities in Limpopo province.

**Public health significance—Why is this work of significance to public health?**

- Provides insight into how the Trajectory of Illness Framework can be adapted to understand chronic disease experiences among rural youths.
- Identifies critical gaps in healthcare delivery, psychosocial support, and disease education for youths living with chronic conditions.

**Public health implications—What are the key implications or messages for practitioners, policy makers and/or researchers in public health?**

- Emphasizes the need for context-specific interventions to improve diabetes management and continuity of care in rural settings.
- Supports the development of youth-centred health policies and integrated care models to enhance long-term outcomes for individuals with Type 1 Diabetes Mellitus.

**Abstract:**

Diabetes Mellitus is a chronic metabolic disorder characterized by elevated blood glucose due to defects in insulin secretion or action, or both, leading to serious short- and long-term complications if not effectively managed. However, there is limited qualitative evidence exploring how youths diagnosed with Type 1 Diabetes Mellitus (T1DM) experience disease onset, management, complications, emotional adaptation, and education within the South African public healthcare system. The study aims to investigate the lived experiences of youths living with T1DM in a selected public hospital in Limpopo province, South Africa. The objectives were to explore and describe the lived experiences of youths living with T1DM. A qualitative, explorative, descriptive, and contextual design was used to gain a thorough understanding of the experiences of youths living with T1DM. A non-probability sampling technique was used to select 12 participants using a pre-determined criterion. Data were collected through individual semi-structured interviews using an interview guide. The data were analyzed using Colaizzi’s method, where themes and sub-themes were developed with the inclusion of an independent coder. Measures to ensure trustworthiness and ethical considerations were adhered to throughout the study. The findings revealed that, despite the participants sharing the same diagnosis, they experience multiple interrelated barriers that significantly hindered effective self-care management, such as limited access to diabetic diet, glucometers and supplies, treatment and informational-related barriers, school-related challenges, transportation constraints and inadequate social support. Furthermore, the findings highlighted gaps in early recognition of symptoms, standardized diabetes education, psychosocial support, and continuity of care. The study recommends the need for holistic, patient-centred, and contextualized interventions that do not only address medical management but the socioeconomic, educational, and psychological needs of youths.

## 1. Introduction

Type 1 Diabetes Mellitus (T1DM) is a chronic autoimmune disorder that profoundly affects the lives of young people during adolescence, a developmental stage characterized by rapid physical growth, emotional maturation, and evolving social dynamics [1,2]. Adolescents living with T1DM frequently experience anxiety and stress related to daily self-management, including insulin administration and glucose monitoring [3,4]. Similarly, youths report feelings of social isolation due to the visible nature of diabetes care routines and dietary restrictions [5,6,7]. Moreover, coping strategies such as reliance on parental guidance or peer support are often essential in helping adolescents manage both medical and psychosocial demands [8].

Internationally, studies in low- and middle-income countries reveal similar challenges for youths with T1DM. In Uganda, adolescents report difficulties accessing insulin and glucose monitoring tools, which increases anxiety and hinders school participation [9,10]. Similarly, Turkish youths describe conflicts between the desire for autonomy and the demands of strict disease management, often resulting in emotional stress [11,12]. Moreover, studies from India highlight that stigma and peer rejection can negatively impact self-esteem and adherence to treatment [13,14,15]. These findings emphasize the universality of the psychosocial challenges faced by adolescents with T1DM across diverse contexts.

Adolescents in high-income countries also experience complex psychosocial dynamics. In the United States, youths report that family conflict and peer pressure often influence adherence to insulin therapy and glucose monitoring [16,17,18]. Similarly, research shows that emotional distress, identity formation, and fear of hypoglycemia affect daily functioning and mental well-being [19,20]. Moreover, adolescents describe a tension between disease management and desire for independence, illustrating how T1DM impacts developmental trajectories [2,21]. These insights reinforce the importance of frameworks that capture both medical and psychosocial aspects of living with T1DM. Interestingly, longitudinal studies suggest that adolescents gradually develop self-management skills over time, transitioning from dependency on caregivers to greater autonomy [22]. Moreover, the presence of supportive social networks, including family, peers, and healthcare providers, is critical in mitigating the emotional burden associated with T1DM [23].

In South Africa, adolescents living with T1DM in rural provinces such as Limpopo face additional structural and socio-economic barriers that compound their experiences [24]. Moreover, adolescents often rely on family and school support to cope with disease management, yet gaps in public health infrastructure impede consistent care [25].

### 1.1. Study Objective

This study aimed to explore and describe the lived experiences of youths living with Type 1 Diabetes Mellitus in the rural communities of Limpopo province, South Africa.

### 1.2. Theoretical Framework

The study adopted Wiener and Dodd Theory of Illness Trajectory to guide the findings [26]. The theory focuses on the individual, the family and the healthcare provider at the centre. The Theory of Illness Trajectory explains living with an illness as a specific type of work that patients carry out with the total organization (family members and healthcare providers) to manage their condition [26]. Although the patient is the primary actor, effective management of the condition also requires the participation of other members of the whole organization. Therefore, the total organization in this study includes patients with T1DM and healthcare providers who work together to ensure stable blood glucose levels by sharing knowledge, support and changing certain aspects of their behaviour.

Wiener and Dodd [26] identified four categories of “work” that people undertake when living with chronic illness: illness-related work, everyday life work, biographical work, and uncertainty work. These categories describe the complicated tasks and adjustments required to manage the disease while preserving the identity and daily functioning.

- Illness-related work

This refers to the practical and medical activities that are directly related to the effective management of the illness. In this context, this refers to the aspects that the youths with T1DM undergo that include diagnosing, identifying signs and symptoms, taking treatments, coping with the emotional reaction to the diagnosis, and dealing with possible side effects. Disease-related work looked at the experiences of youths when they received their diagnosis, their experiences of symptoms and treatment in the clinic and or hospital, and ongoing treatments that they are prescribed. This element focuses on the emotional response of youths when receiving a T1DM diagnosis.

- Everyday life work

This is an effort to maintain everyday routines and responsibilities in spite of the illness. In this study, everyday life work includes activities necessary to accomplish everyday life tasks such as self-care, household and paid employment, family and social relationships, and leisure time [26]. In the context of this research, everyday life work is defined as the self-care work required to manage T1DM, including diet-related practices, taking prescribed medications, adjusting one’s personal and professional lifestyle, and performing daily activities.

- Biographical work

This refers to the method of integrating the illness into one’s life story and identity to be able to merge the illness with one’s idea of self and plans. Within this context, biographical work relates to knowledge of youths with T1DM, including its manifestations, etiology, management (injection sites and techniques), hyper and hypoglycaemia management, as well as its long-term consequences and psychosocial impacts.

- Uncertainty work

**Figure 1:**
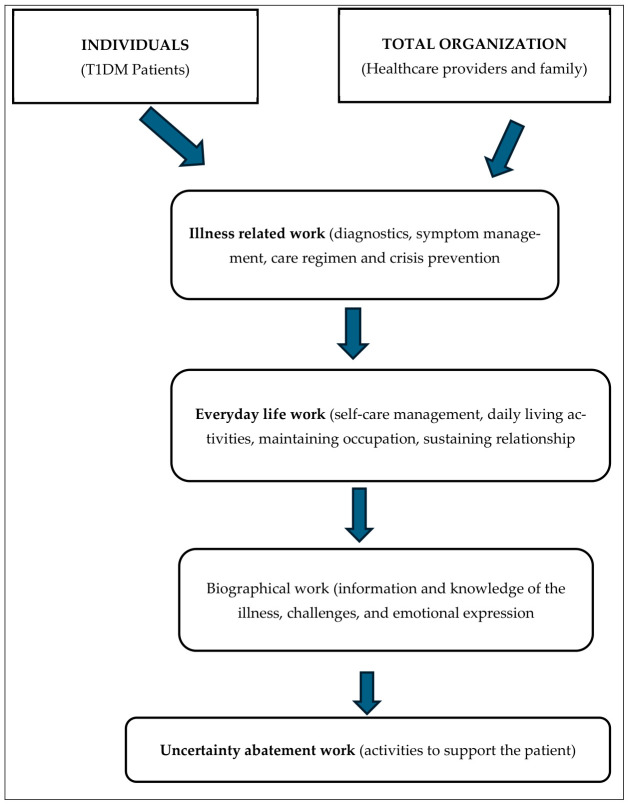
Schematic presentation of the Theory of Illness Trajectory adapted from Wiener and Dodd (1993) [26].

This refers to coping with the unpredictability of the illness and its outcomes to reduce stress and sustain psychological stability. Furthermore, it refers to the activities that an individual performs to reduce the uncertainty associated with a specific illness. In this context, the study underlines how youths diagnosed with T1DM actively journey through the unpredictability of their condition by improving the way in which their illness trajectory is understood. The uncertainty abatement work is the conscious strategies, actions, and practices those individuals undertake to minimize the unpredictability and uncertainty linked with living with a chronic illness. Uncertainty reduction work relates to the activities that an individual performs to reduce the uncertainty associated with a specific illness. This study describes T1DM, which is incorporated into web-based programmes that target work to manage T1DM in youths and is described in detail using the Wiener and Dodd Theory of Illness Trajectory. The schematic presentation of the theoretical framework is shown below in Figure 1.

## 2. Research Methods and Materials

### 2.1. Research Design

This study adopted qualitative, explorative, descriptive research and contextual design to gain a deep understanding of the lived experiences of youths living with T1DM in their natural contexts. This approach allowed researchers to capture rich and detailed accounts of how participants experienced the diagnosis and management of diabetes while living in rural areas of Limpopo province.

### 2.2. Setting

This study was carried out in six selected rural district hospitals in Limpopo province (St Rita’s, Jane Furse, Lebowakgomo, Bela-Bela, FH Odendaal, and Matlala hospitals). Therefore, the selected rural settings were chosen to explore the experiences and challenges related to the management of Type 1 Diabetes Mellitus in rural areas with healthcare challenges.

### 2.3. Participants

The participants were youths living with T1DM, who attended selected hospitals in Limpopo province, South Africa. A non-probability purposive sampling method was used to recruit 12 youths with T1DM from the district hospitals in the Limpopo province. The participants were between 14 and 30 years old, and the rationale for this age group is that the 14- to 18-year-olds are a group transitioning from childhood to adulthood, still managing their illness independently with family support. The 19- to 24-year-olds are emerging adults often considered as youths, and the 25- to 30-year-olds are young adults, where many frameworks, including the African u and UN agencies, still categorize individuals up to 30 years as youths. The participants had lived with TIDM for at least 6 months, which was considered sufficient time to experience the lived realities of the condition. The inclusion criteria consisted of only outpatients who attended hospitals for clinic visits or the collection of prescribed medications.

Although twelve participants might seem to be few, the number of participants was based on data saturation. The sample size in a qualitative study is limited, since the goal is to gain data that aid in comprehending the complexity or context of the phenomena. For a qualitative, explorative and descriptive study, methodologists like Guest et al. (2006) advise a sample size of 3–10 participants [27]. The characteristics of the participants can be seen in Table 1.

### 2.4. Data Collection

The participants were recruited by the researcher, who started by launching a campaign in the villages, high schools, and healthcare clinics to promote the research using flyers and leaflets. The researcher sought permission from the local authorities to conduct campaigns. The healthcare institutions were contacted for a visit during follow-up days for TIDM to recruit participants. The potential candidates were found in the hospital’s outpatient department, where they were purposively selected after explanation of the aims and objectives were explained, an information brochure was issued, and a consent form was signed. Individual semi-structured interviews served as the basis for data collection. The explanation about audio recording of interviews was given, and the rationale was explained with a request for their consent to the recording. A signed assent form was obtained from participants under 18 years, and written informed consent was obtained from their parents before scheduling an interview appointment. The interviews were in English as both participants and the researcher were able to use it during communication. Eleven of the interviews took place in separate rooms in the hospitals that the youths themselves or their families visited, and one interview took place in a young person’s home, when the young person himself/herself had asked to be interviewed at home. Each interview lasted approximately 30–45 min. Every participant had one interview.

The interview guide included open-ended questions followed by probing questions to facilitate data collection. All interviews started with demographic data questions and then proceeded with the main question: “*How do youths living with Type 1 Diabetes Mellitus in rural Limpopo experience and manage their illness over time?*”. This was supported by the following sub-questions, each aligned with specific elements of the Trajectory of Illness Framework:

- Illness onset (trajectory phase):

*How do youths describe their experiences at the time of diagnosis?*

- Illness work (management of the condition):

*How do youths manage their diabetes in their everyday lives?*

- Biographical disruption (impact on identity and daily life):

*How does living with Type 1 Diabetes Mellitus affect their social, educational, and personal lives?*

- Social and environmental context (external influences):

*What role do family, community, and healthcare services play in shaping their experiences?*

- Trajectory management and adaptation over time:

*How do youths cope with and adapt to living with diabetes over time?*

The integration of these framework elements during the data collection phase ensured that the interview questions captured the dynamic and evolving nature of living with a chronic condition, while also attending to the personal, social, and contextual dimensions of the illness experience.

Data collection continued until data saturation was reached. The tenth interview marked the point at which the data were saturated, as participants were reiterating the same information and there were no new ideas or topics brought up. But the researcher conducted two further interviews to rigorously verify the data saturation. The interviews yielded no new insights; the participants reiterated the issues brought up by the initial ten respondents.

Data were collected by the researcher. The researcher was a registered nurse with 10 years’ experience working at the clinic. Providing primary healthcare services to patients with minor ailments, maternal health and follow-up care for all chronic disease patients including diabetes. The researcher had been trained in qualitative data collection techniques. To minimize bias, the researcher used bracketing to avoid including preconceived ideas during interviews

### 2.5. Data Analysis

Data were analyzed using the seven steps of Colaizzi’s data analysis method to establish a fundamental structure of the event [28]. The Colaizzi’s method in this study was used for the purpose of organizing and describing the participants’ experiences, not to establish the in-depth meaning of the findings. This study employs an exploratory and descriptive approach; as a result, Colaizzo’s method was exclusively used to describe the experiences of youths with Type 1 Diabetes Mellitus. While Colaizzo’s method is mostly used for comprehensive interpretation of phenomenological research, its use in this study was exploratory and descriptive.

The researcher and the supervisor read the transcript of each participant’s interview, listened to the audio-taped interview repeatedly, and learned about the participants and their responses; this strategy was employed to familiarize the researcher and the supervisor with the data. The researchers then identified and highlighted all statements in the transcripts that were immediately relevant to the study.

The third stage is that the researchers thoroughly examined the core claims to see which meanings are relevant to the study. This required a constant comparison between the original transcript, the statements, and the participants’ understood meanings. The researchers then gave priority to the participants’ statements and disregarded their own thoughts, perspectives, opinions, and assumptions. Using bracketing, the fourth step was to group the identified meanings into themes that were common across all transcripts. To verify the clusters or themes, the researchers compared them to the original interview and made changes to more accurately represent the participants’ objectives. This required a number of iterative procedures.

At the fifth step, the researchers then created a detailed overview of the events, addressing every issue that was raised in step 4. In six stages, the researchers summarized the lengthy explanation into a concise and all-inclusive claim that highlights the key aspects of the experiences of youths with Type 1 Diabetes Mellitus.

In conclusion, the final step of member checking was conducted to enhance the credibility of the findings. The researchers invited the participants who were present to verify the explanation of the data and determine whether they accurately reflect their experience. Furthermore, validation of the data not only relied on member checking, but it was also dependent on the prolonged engagement with the data, and the involvement of an independent coder who was an expert in qualitative research data analysis, where a consensus was reached regarding the identified themes and sub-themes.

### 2.6. Ethical Considerations

Ethical approval for the study was obtained from the Turfloop Research Ethics Committee of the University of Limpopo (TREC/61/2024: PG). Additional permission was granted for the investigation by the Limpopo Provincial Department of Health, as well as by the district managers and chief executive officers of the participating hospitals.

The rights of the participants were respected throughout the study. They were fully informed about the purpose, objectives, potential benefits, risks, and procedures of the study. Participation was entirely voluntary, and participants were informed of their right to withdraw at any time without penalty. Only participants who met the inclusion criteria were enrolled in the study. Written informed consent was obtained prior to data collection. The study included participants who were below 18 years and above 18 years. Written informed consent was obtained prior to data collection. Those who were older than 18 years signed the informed consent and volunteered to participate in the study. The parents or guardians of the minors gave their informed consent for their children to participant in the study. Furthermore, the researcher obtained a signed assert form from the minors. The researcher explained clearly to the minors regarding the nature and objectives of the study, the expectations and voluntary participation. Anonymity and confidentiality were ensured through the use of pseudonyms in all study records and reports.

### 2.7. Trustworthiness

Trustworthiness in this study was ensured through strategies aligned with Lincoln and Guba’s (1985) criteria [29]. Credibility was achieved by engaging extensively with participants, conducting in-depth interviews, and employing member checking to confirm that the interpretations accurately reflected their lived experiences of Type 1 Diabetes. Dependability was maintained through meticulous documentation of the research process, including sampling, data collection, and thematic analysis using the Trajectory of Illness Framework, allowing for transparency and replication. Transferability was addressed by providing thick descriptions of the rural Limpopo context, participants’ demographics, and the challenges faced in managing diabetes, enabling readers to determine applicability to similar settings. Finally, confirmability was ensured through reflexivity, with the researcher maintaining a reflective journal to monitor personal biases, and through an audit trail that documented all decisions and interpretations, ensuring that findings were grounded in participants’ accounts rather than researcher assumptions.

## 3. Results

The findings are discussed in conjunction with the literature that reinforces the experiences of youths living with Type 1 Diabetes Mellitus in the rural areas of the Limpopo province. The theory of the trajectory of illness by Wiener and Dodd (1993), as found in their four works on illnesses, has been used as the main dimensions [26]. The final themes and sub-themes that serve as the findings of the studies and verbatim comments from the 12 participants are reported next. Themes and subthemes are presented in Table 2 below.

### 3.1. Illness-Related Work Experiences

The first dimension of the theory of the trajectory of illness is the illness-related work. It consists of regimen work, crisis prevention and handling, symptom management, and diagnostic-related work [26]. The participants in this study were asked to describe in detail the symptoms that they were experiencing at the time of diagnosis, how the patients were diagnosed, how the symptoms were managed in the clinic or hospital, and the patients’ feelings and reactions following their diagnosis.

The findings demonstrate that patients living with T1DM experience their condition in a variety of and multifaceted ways, despite sharing the same diagnosis. Differences were evident in the presentation of symptoms, diagnostic pathways, complications, psychological responses, and access to diabetes-related education. These variations highlight the individualized nature of T1DM and underscore the importance of personalized patient-centred care within public health facilities in Limpopo province.

#### 3.1.1. Experience of Diabetes Symptoms

The experiences of diabetes symptoms among participants reflect the illness onset phase of the Trajectory of Illness Framework, which marks the beginning of the illness trajectory and shapes how individuals enter into care. The framework posits that the trajectory is influenced not only by the clinical course of the disease but also by how symptoms are perceived, interpreted, and acted upon. In this study, participants reported a wide spectrum of symptom experiences, ranging from mild to severe. For some, the onset was gradual, characterized by manageable symptoms such as thirst, hunger, and fatigue. This led to delays in seeking care, demonstrating how limited symptom recognition can prolong the early phase of the trajectory. For others, the onset was acute and severe, involving collapse, seizures, or loss of consciousness, which accelerated entry into the healthcare system.

Importantly, the findings show that progression through this phase was not determined solely by symptom severity but also by social factors, such as the involvement of family members or teachers in recognizing symptoms and facilitating care. This illustrates the framework’s emphasis on the interaction between the illness course and contextual influences in shaping the trajectory.

As evidenced by:

*I was really dizzy and vomiting a lot and after a few days started having piles. When I went to the bathroom, I did not have a bowel movement, but instead I was only passing blood (PA).*

*“I started getting weaker every day with frequent hunger and thirst, then started vomiting” (PD).*

*The teacher noticed that she was not feeling well, and before long, she began to shake violently and then collapsed. At that point, they quickly took her to the clinic for medical attention (PE).*

*The girl suddenly threw up a lot and then fell to the ground, so her family quickly took her to the doctor’s office (PK).*

*“I felt hungry even when I ate and drank a lot of water and lost weight” (PC).*

*“My mother said she noticed that my urine was shiny and sticky on the toilet for days, having wet palms recently” (PC).*

#### 3.1.2. Experience of Being Diagnosed with Diabetes

The diagnosis of diabetes represents a critical transition point within the illness trajectory, where individuals move from experiencing symptoms to engaging with formal healthcare systems. According to the Trajectory of Illness Framework, this phase involves the initiation of medical management work aimed at stabilizing the illness and preventing further deterioration. Participants’ accounts demonstrate varied pathways to diagnosis, including both clinic- and hospital-based entry points. Regardless of the setting, diagnosis was consistently triggered by elevated blood glucose levels, confirming the clinical progression of the disease. However, many participants were diagnosed at an advanced stage, requiring urgent interventions such as intravenous fluids, insulin therapy, and hospital admission.

This reflects how delays in the illness onset phase can lead to a more critical trajectory entry point. The immediate medical responses described by participants illustrate the institutional management of the illness, where healthcare providers take primary responsibility for stabilizing the condition. Thus, this sub-theme highlights how the trajectory is shaped by both the timing of diagnosis and the capacity of the healthcare system to respond.

As evidenced by the following:

*I went to the clinic, and the nurse checked me thoroughly. She took my blood pressure, tested my urine, weighed me, and even pricked my finger to check my blood glucose levels. After all the tests, she told me that I have diabetes because my glucose levels were high and it even appeared in my urine (PE).*

*I was in a really bad way, so they put in a drip and called an ambulance. Next thing I knew, I was going to be taken to the hospital. But even with all the medical attention, I was not getting any better at that point (PF).*

*I spent two days in the hospital and had an IV drip throughout the time. I also got three shots every day (PH).*

*I was in the hospital for three days because my blood sugar levels were too high. Doctors and nurses cared for me, giving me fluids by drip and some medication to help control my glucose (PK).*

#### 3.1.3. Experience of Diabetes Complications

The reported complications illustrate the progression of the illness trajectory, and the ongoing challenges associated with illness management (illness work). The Trajectory of Illness Framework emphasizes that living with a chronic condition requires continuous management, and disruptions in this process can lead to instability in the trajectory. Participants described both acute and long-term complications, including diabetic coma, ketoacidosis, and vision loss. These experiences highlight moments where the trajectory becomes unstable due to difficulties in maintaining appropriate blood glucose control. The causes of these disruptions were multifaceted, including inappropriate medication adjustments, non-adherence to treatment, and limited understanding of disease management.

Importantly, some participants expressed uncertainty about the causes of their complications, suggesting gaps in knowledge and self-management skills. This aligns with the framework’s recognition that illness work is not only physical but also cognitive and educational, requiring individuals to understand and actively manage their condition. Thus, this sub-theme demonstrates how the trajectory is continuously negotiated, with periods of stability interrupted by crises when illness work is compromised.

As evidenced by the following:

*I once had a really scary experience with my diabetes treatment. My doctor increased my doses of medication and just two days later I ended up in a diabetic coma because my blood glucose levels had fallen way too low. It was so bad that I had to be admitted to the intensive care unit, which was a very frightening ordeal (PJ).*

*I was hospitalized with ketoacidosis because I hadn’t received the treatment I needed and I didn’t follow my diet (PH).*

*‘I once lost sight for months’ (PL).*

#### 3.1.4. Psychological Experiences Related to Diabetes

The psychological experiences of participants reflect the processes of biographical disruption and adaptation, key components of the Trajectory of Illness Framework. Chronic illness is understood to disrupt an individual’s sense of self, future expectations, and daily life, requiring ongoing psychological adjustment.

Participants described strong emotional reactions at diagnosis, including shock, sadness, and fear, particularly among those with little prior knowledge of diabetes. This illustrates the initial disruption of their life narrative, where the diagnosis represents an unexpected and life-altering event. The presence of a family history of diabetes further shaped these experiences, either intensifying distress or facilitating acceptance. Over time, some participants began to adjust to their condition, indicating movement towards adaptation within the illness trajectory. This process involved coming to terms with the diagnosis and integrating diabetes management into their daily lives. This sub-theme highlights that the illness trajectory is not solely biomedical but also deeply psychosocial, involving continuous negotiation of identity, emotions, and meaning.

As evidenced by the following:

*It was really difficult for me to see my cousin go through a hard time, it hurt me deeply (PK).*

*I was taken aback at first, but as the day went on, I realized that diabetes runs in my family, so it was not entirely unexpected (PG).*

*I was not shocked when I found out, to be honest, because diabetes has been part of my family for a while now (PF).*

*I was taken aback when I heard the news for the first time, it was a real shock to me (PD).*

*I was really sad when I found out that I had diabetes, it was the last thing I expected to happen to me (PH).*

### 3.2. Everyday Life Work Experiences

Everyday life work according to the Theory of Illness Trajectory encompasses the activities of daily living, keeping a household, maintaining an occupation, sustaining relationships, and recreation [26]. Participants in this study were asked to describe the day-to-day self-care management of their condition at home and at school or work. The participants described their daily self-care management in relation to the management of the insulin, monitoring of the glucose, dietary management and the maintenance of occupation and sustaining relationships, as described below.

#### 3.2.1. Management of the Treatment

The patients described their daily self-care management in administering insulin, including the technique of injecting the insulin, the storage of the insulin, the injection sites and the dosage of the insulin prescribed, the side effects of the injection, demonstrating understanding of the subcutaneous insulin administration, and setting an alarm for a reminder.

Participants demonstrated having a knowledge of insulin storage; they highlighted that they store it in a refrigerator. Furthermore, when they go to school, they put their insulin on an ice pack to maintain the cold chain. When the insulin’s colour changed, it was discarded. This highlights that youths understand proper insulin storage to maintain the effectiveness of the treatment.

*“They said I must store them in the fridge and discard them if they change colour” (PB).*

*“When I go to school I use an ice pack, wrap it with a plastic and store in a bag then inject in a toilet” (PB).*

The participants demonstrated understanding of the insulin injecting sites and the importance of changing the injecting sides. Most of them were found to be injecting in the thighs, arm and abdomen; however, there was an area of preference, which was based on the personal comfort or perceived effectiveness.

*“I inject on the thigh, but when I get hurt on the thigh, I then inject the arm” (PA).*

*“I change between my thigh and my abdomen” (PC).*

The youths with T1DM also reflect on the injecting techniques; these include cleaning the injection site, pitching of the skin and then injecting. This demonstrates that the participants had an understanding of how to administer insulin, as advised by the healthcare providers. However, some youths reported feeling pain during injections, scarring and sometimes bleeding. This suggest that insulin injection is physically uncomfortable, which may affect youths’ compliance to the treatment. Furthermore, scarring and bleeding can also suggest incorrect injecting techniques or injecting with one needle several times or localized skin complications.

*“I clean the skin, like I wipe and pinch the skin then inject, it has been for years, but I still feel pain” (PB).*

*“I sometimes have bleeding when I inject, I have scars on the legs but on the arm, I am able to do it nicely” (PA).*

Lastly, the findings also reflect the daily injection schedule followed by the youths, such as injecting insulin 30 min before meals and injecting the short-acting insulin before meals and the long-acting at night. This reflects an understanding of the relationship between insulin administration and meals in controlling the glucose in the body.

*“I inject 30 min before eating, so I, I eat at 6 in the morning, my lunch is at 1, and eat supper at 7, all this time I inject actrapid five units, and only inject protophane six units at 9 p.m.” (PB).*

The findings aligned with the trajectory of illness work, whereby youths with T1DM are actively making decisions and maintain treatment continuity despite the challenges that they are experiencing. These include things like switching up the insulin administration sites, injecting techniques, and proper storage of the insulin. Additionally, the findings demonstrate the continuous adaptation and coping efforts associated with chronic illness management, as highlighted by the Trajectory of Illness Framework.

#### 3.2.2. Glucose Level Management

The participants reflected about their everyday experiences of managing their glucose levels. Although some participants were found to have a glucometer, they did not have adequate glucometer strips to routinely monitor their glucose levels. This implies that the health department is not adequately supporting these young patients’ self-glucose monitoring practices. The glucometer gives youths with T1DM the ability to track their glucose levels on their own, which is essential for controlling their condition. However, the participants emphasized that they monitor their glucose levels prior to eating. This shows that they seem to understand the fundamental glucose monitoring techniques.

*“yes, they gave me the machine on the 4th of October, and I check before meals” (PA).*

*“it happened the other day while I went to check up at the clinic, I did not have food, it was low, it was 2 and they said if it drops to 1 it will be diabetic coma again” (PA).*

The results also shed light on how youths with T1DM cope with low glucose levels. The participants exhibit signs of hypoglycaemia and the treatment thereof. Participants were discovered to be immediately increasing their sugar consumption in order to address their hypoglycaemia by drinking sugar water.

*“I made sugar mixed with water, and it picked up to 3, and they gave me treatment and went back home ” (PA).*

This demonstrates an awareness of the steps to take in an emergency. But one participant without the glucometer emphasized that she could not tell if her blood glucose was low or high, but that she would eat something whenever she felt lightheaded or unable to concentrate, and that 10 min later, she would feel a lot better. Nonetheless, this indicates that a glucometer needs to be provided to all insulin-dependent patients to be able to differentiate the emergency symptoms like hypoglycaemia and hyperglycaemia. Lack of understanding of the signs of a diabetic emergency may lead to an inadequate response.

*“I do not know if it is high or low but whenever I feel somehow like eeeh dizzy or weak I just eat some snack, and I become fine in about 10 min” (PB).*

The findings are aligned with the trajectory of everyday life work because youths with T1DM maintain the stability of their illness by monitoring their glucose, identifying signs of hypoglycaemia and acting accordingly and taking actions. Participants without a glucometer or strips may, however, have a higher risk of uncontrolled diabetes and have their capacity to manage their illness stability affected. Insufficient resources disrupt the effective trajectory management.

#### 3.2.3. Dietary Management

According to the study, participants with T1DM in the Limpopo province adhere to a pre-determined schedule for their meals that complements the timing of their insulin administration. According to one individual, they take their insulin injections and eat at specific times of the day. This indicates that individuals with diabetes have learned the significance of consuming food at the proper intervals in order to maintain their blood glucose levels when using insulin. They also know what foods are beneficial for them, such as bread with low-fat milk, soft porridge, or Weetabix, all of which are advised for controlling diabetes.

*“I eat in the morning, I eat at half past five, at six I inject, at half past five they say I must eat two slices of bread with low-fat milk, and at nine I eat soft porridge or the Weetabix, at twelve I eat porridge, at two I don’t eat, I will eat at half past five, at six I inject and then I will eat at half past seven so that at eight I will inject” (PA).*

According to the findings, youths attempt to adhere to the dietary regimens recommended by medical experts. One participant even claimed to have consumed the foods recommended to them, demonstrating their willingness to adhere to medical guidance and their understanding of how their diet affects their blood glucose levels. They are aware that diet is a crucial component of managing their condition, and they are prepared to modify their habits in order to maintain their health.

*“I eat as they told me” (PA).*

Furthermore, the findings also demonstrate how financial difficulties might impact youths’ dietary choices. Since they do not have a job, one participant stated that they only eat what is at home since they cannot always acquire the food they are supposed to eat. Despite these restrictions, the individual is attempting to follow the advice given by medical professionals to diabetics by eating smaller meals more frequently, such as five or six times daily. Although this is positive, it is obvious that not having enough money might be a significant barrier to maintaining a nutritious diet. Even though they know what they should be eating, they cannot afford those foods, so they end up eating what is available at home. This leads to poor eating habits, which can cause large swings in their blood sugar levels and increase the risk of serious problems such as hyperglycaemia.

*“since I am not working, I just eat what is available at home, but I make sure that I do not eat a lot at once but eat five to six times in a day” (PB).*

When it comes to maintaining a healthy diet, having a supportive family can make a very significant difference. According to one participant, their family members would occasionally buy the recommended meals, but not always. This demonstrates that it can be simpler to adhere to dietary recommendations when all members of the family are on board, even if it is not always achievable due to a lack of resources. It can be quite empowering to have a team behind you supporting you in making positive decisions.

*“yes, most of the food on the list we eat them at home not as drawn, but they are there” (PC).*

*“and at home they are buying the recommended diet for me but not always but most of the time they do” (PC).*

The results emphasize the importance of dietary control in successfully managing the trajectory of chronic diseases like T1DM. The trajectory of everyday work life emphasizes the necessity for patients to have access to vital resources and support in order to properly manage their chronic disease. As a result, youths with T1DM should be encouraged to follow a diabetic diet in order to successfully manage their condition.

#### 3.2.4. Maintaining Occupation and Sustaining a Relationship

The participants revealed that they maintain their work and sustain relationships through strong support from the school or workplace. For youths with T1DM, having a solid support system at school or work appears to be essential. By assisting youths in managing their illness throughout the day, teachers and school employees may have a significant impact. For example, some teachers will go so far as to provide a special cooler with ice packs to maintain the correct temperature of insulin until the afternoon injection. This sort of assistance is essential since it ensures the safety and efficacy of the medication and also demonstrates that the school personnel are concerned about the student’s health and well-being.

*“I am attending school, and for my injection that I get at 2, the teachers gave me a cooler box, and they bring me ice to store my treatment and after school I take my treatment, and every morning they bring ice packs” (PA).*

*“I put my treatment in the managers’ refrigerator” (PD).*

The participant also claimed to have taken insulin in the principal’s office, implying that the school has a private location for this.

*I inject myself in the principal’s office (PA).*

This demonstrates the school’s willingness to support the student’s healthcare requirements, which might help the student feel at home in the classroom and like they belong there.

When youths with diabetes disclose their illness to their employers, they may experience some very positive workplace modifications. For instance, one participant claimed that, after informing their supervisor about their diabetes, they were transferred to another department rather than losing their job.

*I told the manager about my condition and luckily, I was not fired but was then moved to the admin section (PD).*

This demonstrates that people with diabetes can maintain their employment and control their illness if workplaces are accommodating and adaptable. It is all about figuring out how to make it work, and having a helpful boss may make a very significant impact.

However, for some participants, workplace issues made things even harder for youths with these conditions. Participants reported that their employers did not do enough to help them, like not giving them a place to store their insulin or making them work in environments that were bad for their health. In some cases, some participants even lost their jobs because they had to miss work due to illness, which added to their financial and emotional problems. For example:

*“They wouldn’t let me use the fridge, saying it is only for people in charge” (PF).*

*“I was fired because I was frequently sick at work and it was due to hard work” (PG).*

The trajectory of everyday life work evaluates how young people with T1DM keep up with their employment or schoolwork despite their condition, as well as how they maintain their relationships. Since the majority of participants are supported by their school or workplace, which helps them manage their illness, the results of this research are consistent with the framework. However, youths who do not receive the necessary support are affected in their everyday work experiences, which disrupts how a chronic condition like diabetes should be effectively managed.

### 3.3. Biographical Work Experiences

Biographical work according to the trajectory of illness theory consists of the interactions with others, in the gathering and dispersing of information, expressions of concern, caring or anger, and the division of tasks. The participants in this study were asked to describe their sources of information about their condition and the challenges that they encounter when managing their condition. The findings highlight the sources of information that the youths use to gather information about their condition as well as the barriers experienced, such as financial barriers, treatment-related barriers, and psychosocial barriers.

#### 3.3.1. Diabetes Educational Sources and Barriers

According to the findings, it seems like youths with diabetes are not always getting the information they need from healthcare facilities. Participants indicated that they are turning to Google and other online resources to learn more about their condition, which suggests that there are some gaps in the education they are receiving from healthcare providers. While it is good that people are taking the initiative to learn more, relying on online resources can be a problem because it is not always clear if the information is accurate.

Some youths with T1DM are lucky and receive good education from their healthcare providers, like nurses and doctors, and do not need to look elsewhere. But overall, it looks like there is a great deal of variation in how well healthcare facilities are educating people with diabetes. For example, many people are using online resources to supplement their understanding of diabetes, which suggests that they are not getting enough information from their healthcare providers. There are concerns about the accuracy of online information and how well people can understand it.

Some healthcare providers are doing a good job of educating their patients, but it is not consistent across the board. This inconsistency in education can have serious consequences for people with diabetes, and it is something that healthcare facilities need to work on.

*When it comes to completing the basics, I usually start by searching online and then fill in the gaps with information from nurses (PL).*

*When I am unsure about something related to my condition, I usually turn to Google to learn more about it. At the same time, my family members also learn from the information I find, which is really helpful to all of us (PE).*

*I don’t really look for information elsewhere because nurses and doctors are already teaching me everything I need to know about T1DM (PA).*

*I don’t usually look for information because I am the one teaching others about diet, exercise, and treatment, and I work with all the staff at the clinic (PK).*

The study also showed that youths with Type 1 Diabetes and their families did not have all the information they needed about the disease. Many participants were unsure of how to take care of themselves. This lack of knowledge made it difficult for them to make good decisions about their health.

Participants needed more practical advice on how to deal with everyday situations, such as what to eat and how to stay active, to help them better control their condition.

In addition, family members often did not receive the education they needed, which meant they were not prepared to handle Type 1 Diabetes emergencies properly. This lack of knowledge among family members weakened support systems at home and made participants more vulnerable in critical situations, as shown by the following:

*“I have learned that intense exercise can actually help control my blood sugar levels, which is really interesting” (PF).*

*“I am not really sure how much porridge is okay for me to have, apparently it is a pretty small amount” (PG).*

*“My family would not know how to react if I suddenly collapsed, except to rush me to the hospital” (PI).*

The course management of the condition is impacted by insufficient educational support, which results in trajectory instability. Educational barriers can result in poor decision-making, difficulties with self-care management, and noncompliance with medication, all of which are inconsistent with the framework. Additionally, since the family is seen as the primary network for managing T1DM in the trajectory illness theory, the youths with T1DM will not receive meaningful support if they lack sufficient understanding.

#### 3.3.2. Treatment-Related Barriers

Treatment-related challenges were found to negatively influence treatment adherence among youths living with T1DM in the Limpopo province. Youths with T1DM expressed discomfort about informing others about their condition. Participants reported missing insulin doses due to pain associated with injections, lack of privacy in public settings and fear of disclosure of their condition, which leads to skipping the insulin dosages, specifically, when they were travelling or visiting others. These suggest that adherence to insulin can also be affected by psychosocial issues such as lack of privacy, fear of being labelled and being away from the familiar environment. For example, when people have to deal with these challenges, it can be very difficult to manage their diabetes properly, and this can have serious consequences for their health, as evidenced by the following:

*“If my treatment gets messed up in the afternoon, I end up missing those doses and have to wait until I can get to the clinic the next day” (PD).*

*“When I am in pain, I sometimes forget to take my medication” (PE).*

*“When I am visiting someone and staying for a while, I would rather not take my medication than tell them I have diabetes” (PB).*

*“If I travel long distances, I usually miss my doses, I feel like people will see me” (PD).*

According to the trajectory of illness theory, chronic illness needs continuous adherence to medication, monitoring of the symptoms and structured routine. However, the findings shows that there is a tension between (illness work) taking medication and (biographic work) interaction with others. These interrupt the trajectory of care because the participants skip the insulin intentionally for social acceptance and identity rather than illness management.

#### 3.3.3. Psychosocial-Related Barriers

People with T1DM face many emotional struggles that make it difficult for them to take care of themselves. They feel judged and misunderstood by society, making them feel bad for themselves and less motivated to manage their condition. Many people think that diabetes is a fatal disease or that insulin consumption is like being addicted to drugs, adding to feelings of shame, fear, and loneliness. These misconceptions make it even harder for people with T1DM to cope with their condition and take care of themselves. As a result, they often feel isolated and struggle to manage their self-care, which can have serious consequences for their overall health and well-being.

*I get asked if having diabetes makes me less afraid of dying (PJ).*

*When I am carrying my cooler bag, people often give me sympathetic looks and start feeling sorry for me (PI).*

*Some individuals assume I am using drugs when in reality I am just taking my prescribed medication (PH).*

Many people who got diagnosed did not get the help they needed to deal with their emotions and come to terms with what was happening to them. Without regular support from a therapist or counsellor, they struggled to manage their feelings and adjust to life with T1DM. This lack of support made it even harder for them to cope, and it is clear that having someone to talk to and get guidance from would have made a big difference in how they handled their condition. For example, they could have received help with processing their emotions, accepting their diagnosis, and finding ways to live with T1DM in a positive way.

*I never had any interaction with a psychologist, no one ever mentioned one to me (PJ).*

*“I was never referred to a psychologist” (PF).*

*I only met him once when he came to the clinic (PE).*

The biographical work of the trajectory of illness theory emphasizes that an emotional response is associated with a diagnosis of a chronic illness, such as Type 1 Diabetes. For instance, being insulin-dependent for the remainder of one’s life after being diagnosed with Type 1 Diabetes at a young age may have an impact on the psychological well-being of this group. The healthcare professionals are only concerned with the biomedical aspects of the condition and pay no attention to the emotional support of youths with T1DM. As a result, the findings indicate that there is a need to improve the emotional support provided to youths with T1DM in order for them to deal with their illness effectively.

### 3.4. Uncertainty Abatement Work Experiences

Uncertainty abatement work consists of the activities adopted to lessen the impact of the illness on the individual or family. The participants in this study were asked to describe the support that they require to improve the challenges that they encounter on a daily basis. The participants highlighted two important challenges for them and their families such as the financial and informational support.

#### 3.4.1. Financial Support

One participant suggested that grant funds be used to support youths with diabetes in purchasing recommended meals and T1DM management equipment, such as glucose metres. The financial strain caused by the continuous treatment of T1DM, particularly for those with few resources, is emphasized by this statement.

Patients with T1DM require genuine assistance from social and healthcare institutions. Giving out sugar-free food packages through social workers or through clinics when patients pick up their medication is one option. This demonstrates that, despite the understanding of the critical role that diet plays in managing T1DM, many individuals have difficulty obtaining nutritious food due to food insecurity and low income. Because of this, it may be extremely challenging for them to obtain the correct meals, which is an essential aspect of managing their diabetes.

*“I don’t know if it is possible but also all the diabetes patients could get grant money to be able to buy the recommended food and machine” (PB).*

*“and also give us food parcel maybe through the social workers or maybe collect the food same time with the treatment at the clinic so that we can comply to the diet, like they cannot give us them all but at least beans and fish” (PC).*

The findings align with the framework, as the Trajectory of Illness Framework emphasizes the need for a chronic disease to be successfully managed from diagnosis and throughout its course. To manage their illness successfully, youths with T1DM need sufficient financial assistance and resources. The youths with T1DM might not be able to carry out self-care management tasks without the financial assistance. Additionally, the framework’s uncertainty abatement work emphasizes that there must be activities to support youths with T1DM adherence to treatment and reduce the burden of self-care management.

#### 3.4.2. Educational Support

Many youths with T1DM believe they require more support in understanding how to control their T1DM. They want access to knowledge that may help them manage it, and they want to learn more about it. Not only do the patients need to be informed, but their family members as well. Families are able to provide better support at home when they are aware of what is happening. Patients want something to refer to at home, such as written instructions, so they may take care of themselves without having to wait for their condition to deteriorate. They can identify issues early and take action if they have the correct knowledge. This might have a significant impact on their ability to manage their illness and increase their sense of self-assurance. Patients may take charge of their health and get the support they need by educating the entire family.

*“secondly, I would say raise public awareness, and also have educational sessions for families to help us cope” (PB).*

*“I wish they can give us information to have at home so that we can read because sometimes you feel somehow and you just wait to see if you will get worse or not” (PF).*

The findings align with the theory, because information about the illness is one crucial activity that is emphasized by the trajectory of disease theory. Information regarding T1DM needs to be given to youths with T1DM, as well as to their families, in order for them to manage it effectively and for the family to offer the required assistance. The framework’s biographical, daily, and illness work, all of which need sufficient resources and understanding, are necessary for the successful management of Type 1 Diabetes Mellitus. As a result, the educational support form is part of the uncertainty abatement work, which is the trajectory’s supporting work, to educate youths about the disease, diet, glucose monitoring, management of complications, and appropriate insulin usage.

## 4. Discussion

This study’s findings regarding the symptom experiences and illness onset of youths with T1DM align with the literature showing that adolescents often perceive early symptoms as non-specific or mild, which can delay diagnosis and increase risk of acute presentations like diabetic ketoacidosis (DKA). The findings of the study are supported by those of Tuohy et al., who, in a meta-synthesis of qualitative studies on CYPDs with T1DM, found that adolescents often struggled to recognize early signs of the condition, leading to delayed help-seeking and crisis presentation, similar to youths in Limpopo who only entered care when symptoms became severe or were noticed by others [29]. International research further confirms the findings of this current study revealing that the initial crisis phase of diagnosis is experienced as distressing and overwhelming, particularly where symptom recognition is low [30]. In contrast, studies from higher-resource settings suggest that structured screening and higher disease awareness can reduce delays in diagnosis, a difference that highlights the impact of health literacy and access disparities between rural LMIC and urban or developed contexts [29].

Participants’ experiences of engaging with formal healthcare during diagnosis in this study demonstrated similarities with other research showing that acute presentations necessitate urgent care and hospitalization. These findings are like those of another study which revealed that, for example, adolescents in recent T1DM studies emphasized that their first encounters with care were rushed and focused on stabilizing hyperglycaemia, often overshadowing comprehensive education and psychosocial support [31]. Unlike in more organized clinical settings with multidisciplinary care teams, where adolescents report earlier engagement in structured self-management education, this is in contrast with the findings of the study, where the Limpopo cohort’s transition into routine care appeared fragmented and delayed, reflecting health system limitations in resource-constrained settings [32]. Moreover, the finding that many participants required urgent IV fluids or prolonged hospital is in agreement with the evidence of a study conducted in sub-Saharan Africa, which showed higher incidences of DKA at diagnosis compared with high-income countries, reinforcing the importance of improving early detection and education systems [33].

The findings of the study indicated the challenges of ongoing illness management and self-care described by youths in rural Limpopo, and these findings are consistent with findings from global patterns, which concluded that adolescents face complex practical and psychosocial barriers. Furthermore, the findings indicated that qualitative research findings consistently show that youths’ daily management tasks such as insulin administration, glucose monitoring, and dietary regulation are deeply entwined with personal routines, family dynamics, and resource availability [34]. However, unlike some findings from high-income settings where technology (e.g., insulin pumps, apps) can facilitate self-care and reduce burden, the findings of this study indicated that Limpopo participants lacked essential glucometer strips and monitoring tools, mirroring evidence from sub-Saharan contexts where financial and supply constraints hinder effective self-management [35]. This contrasts with findings in South African adolescents with well-controlled T1D, where internal resilience factors and strong external support systems (family, school) helped mitigate barriers [32]. Thus, while fundamental self-management themes are globally consistent, resource constraints significantly differentiate the lived experiences of rural youths [36].

In most of the existing literature [16,21], the financial and educational challenges are consistent. The financial difficulties, though, are context-dependent, and any other situation would be different. The repeated occurrence of these challenges emphasizes the lack of successful government and health system interventions in dealing with youths with Type 1 Diabetes, particularly in rural areas with few resources.

The psychological and social experiences identified in this study, including shock at diagnosis, emotional distress, stigma, and fears related to social judgement, are widely documented across diverse settings. These findings correspond with those of a study conducted in research from Europe and Asia, which indicates that adolescents with T1DM often struggle with identity disruption, fear of peer scrutiny, and worry about future limitations, influencing self-management behaviours and social participation [31]. Furthermore, Zambian youths’ reports of stigma and emotional burden are consistent with these themes, but they also reflect unique rural challenges, such as minimal access to psychological support services and pervasive misconceptions about diabetes within communities [33].

The Trajectory of Illness Framework was applied in this study to explore and understand the lived experiences of youths with T1DM in the rural areas of Limpopo province. The framework guided the organization of data into the phases of illness work, everyday life work, biographical work, and uncertainty abatement, allowing the researchers to capture both biomedical and psychosocial dimensions of living with T1DM [26]. Participants’ experiences of symptom recognition and diagnosis reflected the illness onset and management phases, while their descriptions of insulin administration, glucose monitoring, dietary adherence, and maintaining school or work routines illustrated everyday life work. Emotional reactions, adaptation to family and social environments, and responses to stigma exemplified biographical work, whereas efforts to obtain educational resources and financial support highlighted uncertainty abatement strategies. Applying this framework enabled a structured and holistic analysis of how youths navigated the trajectory of Type 1 Diabetes, demonstrating its suitability for examining complex, chronic illness experiences in a resource-limited rural context.

## 5. Recommendations

Youths with T1DM need to have a good support system, including their family and healthcare providers. They need help managing their condition in a way that works for them. This means getting care that looks at the whole person, not just their medical needs. These include assisting them with assistance regarding school, money, and mental health issues. If healthcare practitioners can provide effective diabetic education and give them emotional support, and the health facilities provide the resources they need, they will be able to take better care of themselves. We healthcare professionals should also try to make their communities and workplaces more supportive for a better health outcome for these youths. This study gives us useful information that can help for policy development, provide better care, and conduct more research to improve the lives of youths with T1DM, especially those who live in rural areas. By doing so, we can make a real difference in their life and help them thrive.

## 6. Strengths and Limitations

The adaptation of the Trajectory of Illness Framework was very helpful in guiding the study, findings and understanding how youths deal with their T1DM illness over time. The qualitative, exploratory, descriptive and contextual study design allowed the researchers to truly dig deep into what it is like for youths living with T1DM. In this way, the youths with T1DM have described in their own words the challenges they face every day and how they take care of themselves. A great deal of detailed and meaningful information that was grounded in their real-life experiences was obtained.

The study also had some weaknesses. For example, the participants in the study had been diagnosed with the disease for different amounts of time, some for just six months, while others had been living with it for 13 years. This could have affected how they felt and what they thought about their experiences, depending on how far along they were with the disease. The study used a self-referral approach to recruit the participants, which can sometimes have some selection bias. However, in this study, the self-referral approach was used to recruit youths with Type 1 Diabetes while protecting their privacy and abiding by ethical standards.

## 7. Conclusions

This study provides a detailed understanding of the experiences of youths living with Type 1 Diabetes Mellitus (T1DM) in rural areas and highlights the complex challenges they face after diagnosis. The findings demonstrate that, although the participants shared the same diagnosis, their T1DM experiences were diverse and shaped by variations in the presentation of symptoms, diagnostic pathways, complications, psychological responses, and access to diabetes education. The study findings found that participants have diverse variations in terms of symptoms, diagnosis, complications, responses and access to health education, and these variations reinforce the view that T1DM is a multifaceted and individualized condition influenced by biological, psychological, and social contexts.

The study revealed that delayed recognition of symptoms and late presentation to healthcare services were common, often resulting in severe clinical manifestations and the need for urgent medical intervention at diagnosis. Following diagnosis, participants encountered persistent barriers that compromised effective self-care management, including financial constraints, treatment-related difficulties, limited knowledge of diabetes, and significant psychosocial stressors. These challenges were further compounded by rural living conditions, inadequate resources, and inconsistent access to structured education and psychological support.

The study therefore highlighted that there are some large gaps in care for youths with Type 1 Diabetes in the rural areas of the Limpopo province. Healthcare practitioners are not doing a very good job of recognizing the symptoms early on, teaching youths and the society about the disease, supporting them emotionally, or making sure they get consistent care. These gaps can lead to some serious problems, such as poor disease management, more complications, and a lower quality of life for patients. It is very important that these challenges be addressed to improve patient outcomes and make diabetes care better in public healthcare settings. The health system needs to do a better job of helping youths with Type 1 Diabetes, and that means filling in these gaps and making sure they get the care they need. Furthermore, investment in rural health infrastructure and school health system collaboration may substantially enhance diabetes self-care and long-term outcomes for affected youths. Longitudinal studies are warranted to further explore how health system and social factors influence diabetes self-management over time.

## Figures and Tables

**Table 1:** Characteristics of the participants.

Participants’ Pseudonyms	Gender	Age	Educational Level	Year Diagnosis	Duration of Diagnosis	Location
Participant A	Female	17	Grade 10	2024	10 months	Ga-Chuene
Participant B	Male	19	Matric	2020	4 years	Ga Masha
Participant C	Male	16	Grade 11	2023	1 year	Ga Rantho
Participant D	Female	29	Tertiary level	2024	6 months	Sevenstad
Participant E	Female	18	Matric	2022	2 years	Morarela
Participant F	Female	15	Grade 8	2019	5 years	Marble hall
Participant G	Male	30	Degree	2018	6 years	Marulaneng
Participant H	Female	24	Matric	2024	3 months	Mahwelereng
Participant I	Female	27	Tertiary	2021	3 years	Ngoabe
Participant J	Male	30	Matric	2011	13 years	Malebitsa
Participant K	Male	23	Degree	2022	2 years	Glen Cowie
Participant L	Male	14	Grade 8	2019	5 years	Ga Mphahlele

**Table 2:** Findings guided by the trajectory of illness theory.

Theme	Sub-Themes
3.1 Illness-related work experiences	3.1.1 Diabetes Symptoms experiences 3.1.2 Diagnosis experiences 3.1.3 Complications experiences 3.1.4 Psychological experiences
3.2 Everyday life work experiences	3.2.1 Treatment management 3.2.2 Glucose level management 3.2.3 Dietary management 3.2.4 Maintaining occupation and sustaining a relationship
3.3 Biographical work experiences	3.3.1 Diabetic Educational Sources and Barriers 3.3.2 Treatment-related barriers 3.3.3 Psychosocial barriers
3.4 Uncertainty abatement work experiences	3.4.1 Financial support 3.4.2 Educational support

## Data Availability

Data supporting the findings of this study are available upon reasonable request from the corresponding author. As data may contain information that jeopardizes the privacy of research participants, they are not publicly available.

## References

[1] Chiang J.L., Maahs D.M., Garvey K.C., Hood K.K., Laffel L.M., Weinzimer S.A., Wolfsdorf J.I., Schatz D. (2018). Type 1 diabetes in children and adolescents: A position statement by the American Diabetes Association. Diabetes Care.

[2] Hilliard M.E., Wu Y.P., Rausch J., Dolan L.M., Hood K.K. (2013). Predictors of deteriorations in diabetes management and control in adolescents with type 1 diabetes. J. Adolesc. Health.

[3] Rechenberg K., Whittemore R., Holland M., Grey M. (2017). General and diabetes-specific stress in adolescents with type 1 diabetes. Diabetes Res. Clin. Pract..

[4] Monaghan M., Helgeson V., Wiebe D. (2015). Type 1 diabetes in young adulthood. Curr. Diabetes Rev..

[5] Scholes C., Mandleco B., Roper S., Dearing K., Dyches T., Freeborn D. (2013). A qualitative study of young people’s perspectives of living with type 1 diabetes: Do perceptions vary by levels of metabolic control?. J. Adv. Nurs..

[6] Freeborn D., Dyches T., Roper S.O., Mandleco B. (2013). Identifying challenges of living with type 1 diabetes: Child and youth perspectives. J. Clin. Nurs..

[7] Hagger V., Hendrieckx C., Sturt J., Skinner T.C., Speight J. (2016). Diabetes distress among adolescents with type 1 diabetes: A systematic review. Curr. Diabetes Rep..

[8] Ahola A.J., Saraheimo M., Freese R., Mäkimattila S., Forsblom C., Groop P.-H., FinnDiane Study Group (2016). Fear of hypoglycaemia and self-management in type 1 diabetes. J. Clin. Transl. Endocrinol..

[9] Beran D., Yudkin J.S., De Courten M. (2005). Access to care for patients with insulin-requiring diabetes in developing countries: Case studies of Mozambique and Zambia. Diabetes Care.

[10] Njabou Katte J.C. (2023). Type 1 Diabetes in Sub-Saharan Africa: Understanding Aetiology and Survival. Ph.D. Thesis.

[11] Yetim A., Alikaşifoğlu M., Baş F., Eliaçık K., Çığ G., Erginöz E., Ercan O., Bundak R. (2018). Glycemic control and health behaviors in adolescents with type 1 diabetes. Turk. J. Pediatr..

[12] Efe Y.S., Erdem E. (2018). A comparison of aggression and self-injury among type 1 diabetic and healthy adolescents: A sample from Turkey. Arch. Psychiatr. Nurs..

[13] Chatterjee S., Bakhla A.K., Biswas P., Singha S., Dubey S., Sharma C.B., Chowdhury S. (2020). Psychosocial morbidity among children with type-1 diabetes mellitus. J. Fam. Med. Prim. Care.

[14] Kumar N., Singh Y., Singh S., Rana V. (2020). Quality of life of type 1 diabetic Indian children and adolescents-Cross sectional study. Int. J. Health Sci. Res..

[15] Capistrant B., Friedemann-Sánchez G., Pendsey S. (2019). Diabetes stigma, parent depressive symptoms and Type-1 diabetes glycemic control in India. Soc. Work Health Care.

[16] Naughton M.J., Ruggiero A.M., Lawrence J.M., Imperatore G., Klingensmith G.J., Waitzfelder B., McKeown R.E., Standiford D.A., Liese A.D., Loots B. (2008). Health-related quality of life of children and adolescents with type 1 or type 2 diabetes mellitus: SEARCH for Diabetes in Youth Study. Arch. Pediatr. Adolesc. Med..

[17] Hood K.K., Peterson C.M., Rohan J.M., Drotar D. (2009). Association between adherence and glycemic control in pediatric type 1 diabetes: A meta-analysis. Pediatrics.

[18] Wysocki T., Harris M.A., Buckloh L.M., Mertlich D., Lochrie A.S., Mauras N., White N.H. (2007). Randomized trial of behavioral family systems therapy for diabetes: Maintenance of effects on diabetes outcomes in adolescents. Diabetes Care.

[19] Fisher L., Hessler D.M., Polonsky W.H., Mullan J. (2012). When is diabetes distress clinically meaningful? Establishing cut points for the Diabetes Distress Scale. Diabetes Care.

[20] Rankin D., Harden J., Waugh N., Noyes K., Barnard K.D., Lawton J. (2016). Parents’ information and support needs when their child is diagnosed with type 1 diabetes: A qualitative study. Health Expect..

[21] Patton S.R., Clements M.A., Fridlington A., Cohoon C., Turpin A.L., DeLurgio S.A. (2013). Frequency of mealtime insulin bolus as a proxy measure of adherence for children and youths with type 1 diabetes mellitus. Diabetes Technol. Ther..

[22] Haugstvedt A., Wentzel-Larsen T., Rokne B., Graue M. (2011). Perceived family burden and emotional distress: Similarities and differences between mothers and fathers of children with type 1 diabetes in a population-based study. Pediatr. Diabetes.

[23] Pacaud D., Yale J.F., Stephure D., Trussell R., Davies H.D. (2005). Problems in transition from pediatric care to adult care for individuals with diabetes. Can. J. Diabetes.

[24] Oosthuizen S. (2024). The Experiences of Young People Living with Type 1 Diabetes in the Western Cape Public Health System: A Thematic Analysis. Ph.D. Thesis.

[25] Fakudze S., Mamba W. (2025). Experiences and coping strategies of adolescents living with Type 1 Diabetes Mellitus: A Descriptive phenomenological study. Nurs. Health Sci. J..

[26] Wiener C.L., Dodd M.J. (1993). Coping amid uncertainty: An illness trajectory perspective. Sch. Inq. Nurs. Pract..

[27] Guest G., Bunce A., Johnson L. (2006). How many interviews are enough? An experiment with data saturation and variability. Field Methods.

[28] Morrow R., Rodriguez A., King N. (2015). Colaizzi’s descriptive phenomenological method. Psychologist.

[29] Lincoln Y., Guba E. (1985). Naturalistic Inquiry.

[30] Tuohy E., Rawdon C., Gallagher P., Glacken M., Murphy N., Swallow V., Lambert V. (2019). Children and young people’s experiences and perceptions of self-management of type 1 diabetes: A qualitative meta-synthesis. Health Psychol. Open.

[31] Montali L., Zulato E., Cornara M., Ausili D., Luciani M. (2022). Barriers and facilitators of type 1 diabetes self-care in adolescents and young adults. J. Pediatr. Nurs..

[32] Yildiz G.K., Besirik S.A., Azak M. (2026). From Diagnosis To Daily Life: Adolescents Navigating Psychosocial Challenges in Type 1 Diabetes–A Qualitative Study. J. Child Fam. Stud..

[33] Mabizela S., Deacon E., Van Rensburg E., Bekker C.I. (2025). Protective factors in resilient South African youth with type 1 diabetes: A qualitative study. Afr. J. Prim. Health Care Fam. Med..

[34] Hapunda G., Abubakar A., van de Vijver F., Pouwer F. (2015). Living with type 1 diabetes is challenging for Zambian adolescents: Qualitative data on stress, coping with stress and quality of care and life. BMC Endocr. Disord..

[35] Hung L.-C., Huang C.-Y., Lo F.-S., Cheng S.-F. (2020). The self-management experiences of adolescents with type 1 diabetes: A descriptive phenomenology study. Int. J. Environ. Res. Public Health.

[36] Desse T.A., Namara K.M., Manias E. (2024). Patient-perceived challenges to type 2 diabetes self-management in sub-Saharan Africa: A qualitative exploratory study. Sci. Diabetes Self-Manag. Care.

